# Percutaneous insertion of bilateral double J stent

**DOI:** 10.1590/0100-3984.2017.0230

**Published:** 2019

**Authors:** Thiago Franchi Nunes, Tiago Kojun Tibana, Rômulo Florêncio Tristão Santos, Jorge da Costa Carramanho Junior, Edson Marchiori

**Affiliations:** 1 Universidade Federal de Mato Grosso do Sul (UFMS), Campo Grande, MS, Brazil.; 2 Universidade Federal do Rio de Janeiro (UFRJ), Rio de Janeiro, RJ, Brazil.

## INTRODUCTION

A number of different techniques and devices can be used for drainage of the urinary tract, including double J stent insertion (cystoscopic retrograde insertion or percutaneous antegrade insertion) and percutaneous nephrostomy^(^^1^^)^.

Double J stenting restores physiological urinary drainage, precluding the need for an external catheter^(^^2^^)^. The typically treatment for ureteral obstruction is retrograde insertion of catheters under cystoscopic guidance^(^^3^^)^. However, the procedure may be unsuccessful or contraindicated in up to 50% of the cases, especially when there is distal obstruction or extrinsic compression of the ureter by a tumor or tumors^(^^4^^)^. Under such circumstances, the patient is typically referred for percutaneous drainage^(^^3^^)^.

Percutaneous insertion of a double J stent under ultrasound and fluoroscopy guidance can be a viable alternative to percutaneous nephrostomy and retrograde catheterization in patients with dilatation of the renal collecting system. Although the technique has a high success rate, its use is still quite limited. Percutaneous insertion of a double J stent has a low potential for complications, and, because it is performed under local anesthesia and sedation, it also minimizes the risk of adverse events related to general anesthesia, especially in critically ill patients^(^^5^^)^. Ideally, it should be performed by interventional radiologists trained in percutaneous procedures.

## PROCEDURE

**Figure 1:**
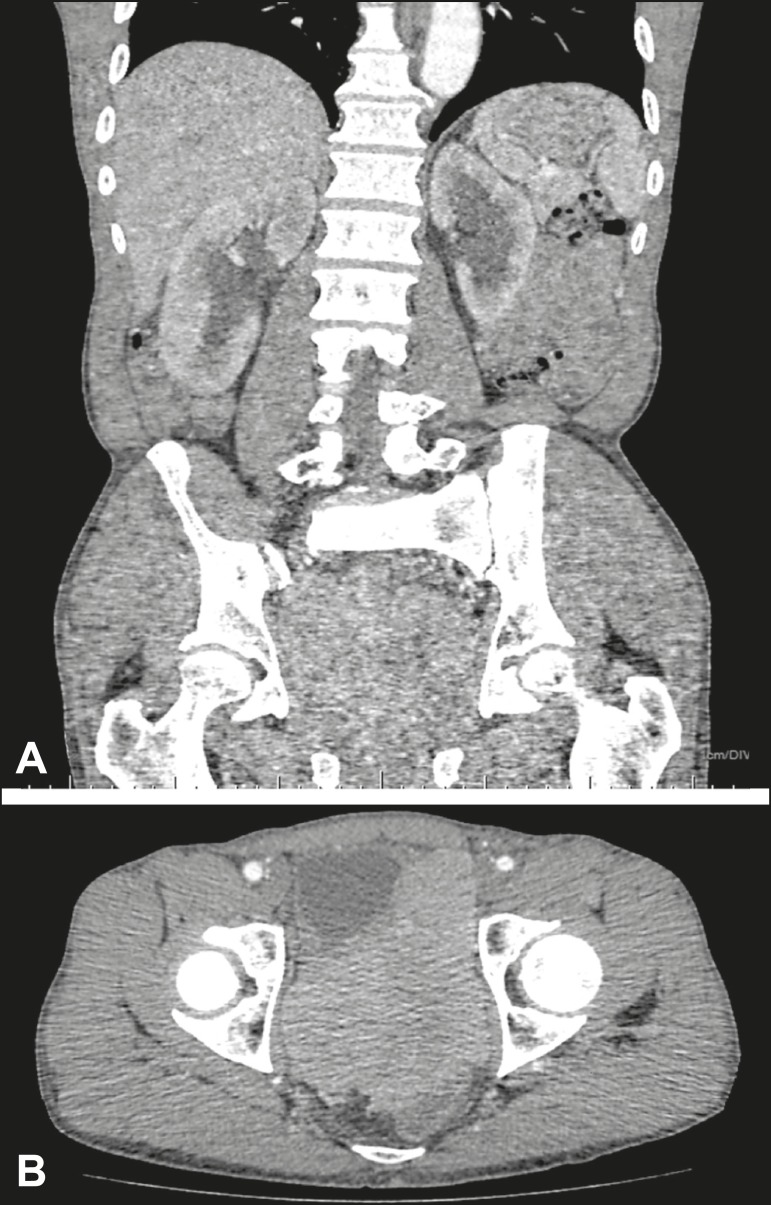
Contrast-enhanced coronal computed tomography (**A**) showing intense bilateral hydronephrosis. Axial view of the pelvis (**B**) showing a mass in the prostatic capsule (sarcoma) invading both ureteral orifices, rendering cystoscopic double J stenting impossible.

Reviewing the imaging examinations prior to the procedure is of paramount importance in the search for findings that contribute to achieving technical success, such as better visualization of the renal collecting system and identification of any potential risk factors for the occurrence of adverse events (Figure 1), such as interposition of the colon between the flank and the kidney.

**Figure 2:**
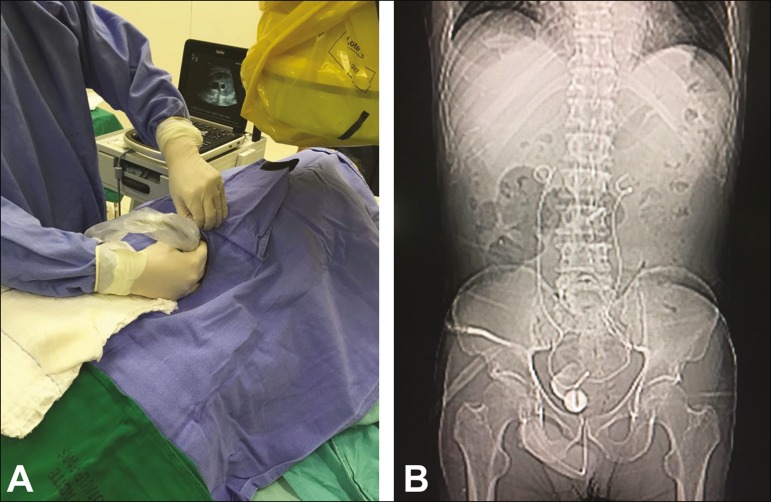
**A:** Ultrasound of the urinary tract being performed in the hemodynamic room in preparation for the percutaneous insertion of the double J stent. **B:** Correct positioning of both double J stents at the end of the procedure.

For percutaneous access to the collecting system, the patient is usually placed in the supine position. The procedure is guided by ultrasound and uses an echogenic needle (Figure 2A), enabling visualization from the moment the needle penetrates the skin until it reaches the renal calyx. The preferred route is through the middle calyx, which offers easier access to the ureteropelvic junction. Another possibility is to go through the lower pole calyx, in a posterolateral approach, which provides a safe and relatively avascular puncture route^(^^3^^)^, which can minimize the risk of complications such as bleeding and pneumothorax^(^^6^^)^. In cases of mild dilation of the collecting system, the coaxial technique, which uses a micropuncture kit, is preferred. Antegrade pyelography is performed with iodinated contrast injection and fluoroscopic visualization of the collecting system.

Once access has been established, a hydrophilic guidewire and a 5F diagnostic catheter are introduced, under fluoroscopic guidance, from the collecting system to the bladder. A 4-mm balloon dilator can be used in order to dilate the ureter when there is any difficulty in passing the catheter through the stricture^(^^3^^)^. A 7F × 45 cm introducer sheath is then positioned and the guidewire and 5F catheter are removed. The double J stent is then passed through the introducer sheath with the help of a J-tipped Teflon-coated guidewire.

At first, the proximal pigtail of the ureteral stent might not be formed. However, follow-up images usually show that the pigtail forms over a few days. We performed an abdominal X-ray at 12-72 h after the procedure to visualize stent positioning and excretion of the contrast medium (Figure 2B).
